# Beyond Singular Climatic Variables—Identifying the Dynamics of Wholesome Thermo-Physiological Factors for Existing/Future Human Thermal Comfort during Hot Dry Mediterranean Summers

**DOI:** 10.3390/ijerph15112362

**Published:** 2018-10-25

**Authors:** Andre Santos Nouri, Ioannis Charalampopoulos, Andreas Matzarakis

**Affiliations:** 1 University of Lisbon, Faculty of Architecture, CIAUD—Research Centre for Architecture, Urbanism and Design, Rua Sá Nogeuira, Pólo Universitário do Alto da Ajuda, 1349-063 Lisbon, Portugal; 2 Laboratory of General and Agricultural Meteorology, Agricultural University of Athens, 118 55 Athens, Greece; icharalamp@aua.gr; 3 Research Centre Human Biometeorology, German Meteorological Service, D-79104 Freiburg, Germany; Andreas.Matzarakis@dwd.de; 4 Chair of Environmental Meteorology, Faculty of Environment and Natural Resources, Albert-Ludwigs-University, D-79085 Freiburg, Germany

**Keywords:** human thermal comfort, physiologically equivalent temperature, urban canyon cases, climate change, Mediterranean climate

## Abstract

Centered on hot dry Mediterranean summer climates, this study assesses the climatic data that was extracted from Lisbon’s meteorological station between the years of 2012 and 2016. Focused on the summer period, existing outdoor human thermal comfort levels that are already prone to extreme heat stress thresholds were evaluated. Such an assessment was rooted around identifying the relationship and discrepancies between singular climatic variables (e.g., air Temperature (T_a_)); and adapted thermos-physiological indices (e.g., the modified physiologically equivalent temperature (mPET)), which also consider the influence of radiation fluxes over the human body. In addition, default urban canyon case studies (UCCs) were utilized to supplement how both differ and influence one another, especially under extreme weather conditions including heat waves events (HWE), and very hot days (VHD). Through the use of wholesome thermo-physiological indices, the study revealed that while human health and thermal comfort is already prone to extreme physiological stress (PS) grades during one of the hottest months of the year, the current extremes could be drastically surpassed by the end of the century. Within the examined UCCs, it was identified that the projected PET could reach values of 58.3 °C under a projected climate change RCP8.5/SRES A1FI scenario. Similarly, and in terms of thermo-physiological stress loads, the following could happen: (i) a future “cooler summer day” could present similar conditions to those currently found during a ‘typical summer day; (ii) a future ‘typical summer day’ could present hourly physiological equivalent temperature load (PETL) that recurrently surpassed those currently found during a “very hot day”; and, (iii) a future “very hot day” could reveal severe hourly PETL values that reached 35.1 units beyond the established “no thermal stress” class.

## 1. Introduction

Within the international arena, the effects of climate change are continually becoming a pressing issue for human health and comfort within consolidated urban environments. During 2018, the occurrence of copious extreme climatic events within the Northern hemisphere was accompanied by topical research suggesting that these climatic manifestations would both continue, and increase, during the next four years [1]. Similarly, the recorded global average air temperature for 2017 rendered it the third warmest year in the National Oceanic and Atmospheric Administration’s 138-year climate record, behind 2016 and 2015 successively [2]. Furthermore, it was argued that for 2017, (i) there was clear evidence of human influence on summer air temperature, both in the overall recordings, and in the heatwave labelled as “Lucifer”; and, (ii) the effects of climate change made the summer of 2017 at least ten times more likely in comparison to the early 1900’s effects [3].

Previously in 2003, and in the case of Western Europe, it became clear that European countries, including Portugal, required additional measures to caution, manage, and diminish recurrences of these events [4,5,6,7]. More specifically for the case of Lisbon, between 29 July and 13 August 2003, the following was identified by Nogueira et al. [8]: (i) 15 days had a maximum recorded air temperature (T_a_) above 32 °C; (ii) there was a noteworthy consecutive run of 10 ten days with a maximum recorded T_a_ above 32 °C; (iii) there was a five day period that consecutively recorded T_a_ values above 35 °C. In association, the research also identified that, as a result of this particular summer period, there was a serious impact on public health, whereby the estimated mortality rate increased by 37.7% (i.e., corresponding to 1316 excess deaths) in comparison to standard/expected figures.

Based on the risk factors associated with human thermo-physiological conditions, interdisciplinary practices including that of urban climatology and biometeorology are also striving to understand how local bottom-up assessments of outdoor environments can lead to a comprehension of both existing and future threats to human thermal comfort thresholds [9,10,11,12,13,14,15,16,17,18,19,20,21,22]. Considering these thresholds, and as reinforced by numerous recent studies [7,23,24,25], the human thermo-physiological perception of thermal conditions exceeds that of solely T_a_, and conglomerates with numerous other imperative climatic variables. Within these studies, it was demonstrated that to effectively evaluate the influence of the thermal environment on individuals, it was necessary to use thermal indices centered on the energy balance of the human body [26]. Of the numerous existing indices, the physiologically equivalent temperature (PET) [9,27] (based on the Munich energy-balance model for individuals (MEMI) [28,29]) is one of the most widely used steady-state models in bioclimatic studies [30].

Regardless of frequent political attritions and future uncertainty, which are concomitant with the topic of climate change, outputs from climatic top-down assessments have thus far identified that global T_a_ will likely continue to increase throughout the 21st century, and that there will moreover be changes in global humidity, wind speed, and cloud cover. Nevertheless, scientific dissemination like the reports presented by the Intergovernmental Panel on Climate Change have frequently described the effect of weather with a simpler index based on amalgamations of T_a_ and relative humidity (RH). Although it is inarguable that those disseminations have significantly propelled the maturing top-down climate change adaptation agenda, when considering bottom-up approaches to climatic vulnerability, the exclusion of vital meteorological factors (i.e., radiation fluxes, wind speed (henceforth expressed as *V*), and human thermo-physiological factors) have arguably diminished the relevance and applicability of the results for local action and decision making [31,32].

Subsequently, the assessments of human thermal comfort conditions need to accompany the growing responsibility for local scales also to tackle the “the high-frequency and microscale climatic phenomena created within the anthropogenic environment of the city” [33] (p. 126). Nevertheless, and as identified by numerous studies [22,24,34,35], the scientific community has already recognized a weakness in the studies that examine local approaches to human thermal comfort thresholds within Mediterranean climates. Similarly, the number of studies that consider the important effects (and possible mitigation efforts) of urban heat islands (UHI) upon local thermal conditions (including for Mediterranean climates) have also grown within the international scientific community (e.g., [36,37,38,39,40,41,42,43,44,45,46]).

To this end, and based on contributing towards methodical means to assess local human health and thermal comfort conditions in climates with hot dry Mediterranean summers, this study examined the following: (i) the relationship between singular climatic variables and thermo-physiological indices; (ii) impact of selected urban morphological compositions on local thermo-physiological indices to identify the crucial role of radiation fluxes, which are intrinsic to the urban energy balance [47]; (iii) synoptic aggravations of current local human thermal risk factors given the occurrence of a potential worst-case-scenario of climate change; and lastly, (iv) intensity and periodicity between current conditions and those projected for the end of the century were cross-examined.

## 2. Materials and Methods

### 2.1. Site

Located on the western coast of Portugal at 38°42′ N and 9°08′ W, the capital city of Lisbon witnesses a climatic Köppen Geiger (KG) classification of ‘*Csa*’ (hot-mediterranean climate), entailing its vulnerability to hot dry Mediterranean summers [48]. As described by Miranda [49] and Calheiros [50], Lisbon presents the following: (i) between 10 and 20 “very hot days” (VHD), where the maximum recorded T_a_ surpass 35 °C; (ii) a range between 100 and 120 “typical summer days”, where the maximum recorded T_a_ surpassed that of 25 °C; and finally, (iii) repeated heat wave events (HWE), where T_a_ consecutively surpass that of 32 °C throughout numerous days. In addition to these factors, the study is based on Lisbon’s historical quarter “Baixa Chiado”, which, due to its morphological composition, often experiences the highest UHI intensities [51] and temperatures during the summer [52].

### 2.2. Data

To acquire the data required for the study, meteorological recordings were obtained from the World Meteorological Organization (WMO) weather station located in Lisbon, with the index N°08535. Similar to numerous studies [17,32,35,53,54,55,56], the retrieved information was then processed through the RayMan Pro^©^ model (RayMan Pro, Research Center Human Biometeorology; Freiburg, Germany; http://www.urbanclimate.net/rayman/) [57,58,59] to determine the PET index [9], and the recently modified PET (mPET) index [60].

Based on the MEMI model [28,29], the decision for utilizing PET was because of the following: (i) calibration on easily obtainable microclimatic characteristics; and (ii) usage of °C as the measuring unit to assess thermal comfort, which in turn, facilitates other professionals to more effectively comprehend and approach urban climatological aspects. In addition, and according to the study conducted by Matzarakis [17], the outputs could subsequently be compared to different grades of human thermal perception and physiological stress (PS), as shown in Table 1.

With regards to the mPET index, and in accordance with Chen and Matzarakis [60], the major differences from the original index are the following: (1) an integrated multiple-segment thermoregulation model; and (2) a clothing model that provides a more accurate analysis of the human bio-heat transfer mechanism. Accordingly, and in comparison to the simpler two-node body model of the PET index, the body model of the mPET index can more efficiently identify heat transfer between the inner body and outer body. Within the specified study that considered bioclimatic conditions in Freiburg during the summer period, the following was verified: (i) when comparing the results against the PS grades, unlike PET outcomes, almost no “extreme heat stress” periods were presented; and, (ii) overall, the probability of comfortable thermal conditions (i.e., of “no thermal stress”) was higher with the application of the mPET index. In line with these outputs, analogous results were also obtained by the authors of [22,23,62], particularly during periods of more accentuated thermal stimuli. 

With regards to singular climatic variables, the following hourly data was collected between the hours of 09:00 and 18:00 from the specified meteorological station: total cloud octas, T_a_, RH, and lastly, *V*. When approaching the latter, once the values of *V* were translated into m/s, a further modification was undertaken to account for the type of urban typology discussed by the study. As presented by Oliveira, Andrade, and Vaz [41], when approaching speeds beneath the urban canopy layer, and within the streets themselves, *V* values are considerably slower than those presented by the meteorological station. Consequently, to determine the actual *V*, which directly influence the gravity center of the human body, as specified by Kuttler [63]; the results from the meteorological station were adapted to a height of 1.1 m (henceforth expressed as *V*_1.1_). Similarly to the studies conducted by the authors of [19,22,23], the formula presented in the literature [63,64] was used (Equation (1)), as follows:

(1) $$\;V_{1.1}=\;V_{h}^{*}{\left(\frac{1.1}{h}\right)}^{\alpha }\;\alpha =0.12^{*}z_{0}+0.18\;$$

where *V_h_* is the m/s at the height of *h* (10 m); *α* is an empirical exponent, depending on urban surface roughness; and *Z*_0_ (m) is the corresponding roughness length.

Comparable to the morphological layout within the study of Algeciras and Matzarakis [19] in Barcelona, and moreover, identical to Nouri, Costa, and Matzarakis [22], and Nouri, Fröhlich, Silva, and Matzarakis [23] who also undertook their study in Lisbon’s historical center, the variables of *Z*_0_ and *α* were calibrated to 1.0 and 0.35, respectively. In addition to these climatological aspects, the calibration of the RayMan model was based on the default standing “standardized man” (equating to a height of 1.75 m, a weight of 75 kg, the age of 35, a heat transfer of clothing (clo) of 0.90, and an internal heat production of 80 watts) [9,27].

### 2.3. Applied Methodology and Structure

The undertaken study was divided into four sequential stages to evaluate the relationship between the singular climatic variables and the thermo-physiological factors of human comfort in an era of potential climate change. Throughout the majority of the study, and in order to facilitate the reading and interpretation of the data, (i) heatmaps were prepared through the use of R language scripts, enabling the climatic data to be displayed through the combination of raster and contour maps assembled by the ggplot2 R language package (ggplot2, Houston, TX, USA, https://ggplot2.tidyverse.org/) [65], and, (ii) the climate tourism/transfer information scheme (CTIS) model (Research Center Human Biometeorology; Freiburg, Germany; http://www.urbanclimate.net/climtour/mainframe_tools_ctis.htm) [66,67] used in similar climatic studies [16,22,54,55,68,69,70].

- Within the first section, and launching the study, the four singular climatic variables for the month of July were assessed. The analysis was carried out between the years 2012 and 2016, to determine the yearly trends and hourly oscillations during the mid-summer period. Once undertaken, the RayMan model was subsequently utilized to obtain both the PET and mPET values, thus enabling the identification of PS thresholds (Table 1).
- Considering the data obtained for 2016, the second section of the study examined three different days during July, based on their overall diurnal climatic conditions. The data was then translated into urban canyon cases (UCCs) to evaluate how existing climatic conditions could vary amongst the different selected urban morphological compositions and their interior regions.
- Within the third section, referring to the data obtained for each UCC, and considering the worst-case-scenarios of climate change, the synoptic projections of human thermal conditions for the end of the century were evaluated. Although indicative, the exercise permitted the study to obtain an initial reflection on how current conditions within different UCCs could modify until the end of the century.
- Lastly, through an optimal benchmark of human thermal comfort, additional adaptations of the base PET index were utilized to compare both the intensity and periodicity of the thermal stress. As a result, both the present and synoptic projections of future thermal environments could be evaluated against one another.

#### 2.3.1. Variable Oscillation for July

In this section, the four singular variables (Octas, T_a_, RH, and *V*_1.1_), which were retrieved from Lisbon’s meteorological station for the month of July were collected, investigated, and presented through the ggplot2 R language package and CTIS model. With the interest of attaining a more comprehensive understanding of July’s climatic conditions, and to moreover account for annual variations, a total of five datasets were processed (i.e., |Jul_2012_|, |Jul_2013_|, |Jul_2014_|, |Jul_2015_|, |Jul_2016_|) for each variable. In the case of T_a_, and referring to the specifications of Miranda [49] and Calheiros [50], it was possible to identify the occurrence of extreme weather occurrences, including VHDs and HWEs, and moreover, inspect how the other three variables interplayed during the events. In addition, as each variable was recorded every hour between 09:00 and 18:00, the outputs presented gradual or abrupt diurnal oscillations, which revealed the hours where certain variables tended to be higher or lower. 

To obtain the PET and mPET indices, the four singular variables were processed through RayMan for each dataset. Within ggplot2 and CTIS, the grades illustrated in Table 1 were used to associate each specific value to a PS classification, as stipulated by Matzarakis, Mayer, and Iziomon [61]. Moreover, as with the presentation of the singular variables, the periods in which the meteorological station underwent calibration (CAL.) were also acknowledged.

#### 2.3.2. Configuring the UCC Assessments

Considering the general morphological composition within Lisbon’s historical quarter, known for its accentuated susceptibility to thermal stimuli during the summer, namely to UHI [51] and elevated T_a_ [52], a canyon height of 20 m remained as the constant physical variable. Contrariwise, and centered on some of the most common street/canyon widths [22], this variable was set to vary between 10, 20, and 80 m. Finally, and although the canyon length is unrelated to the height-to-width (H/W) ratio, each canyon was configured to a length of 200 m, to ensure that the “edges” of the canyon would not influence the obtained results. Consequently, and through “Obstacle Plugin” within the RayMan model, three UCCs were configured with varying height-to-width (H/W) ratios (Table 2).

**Figure 1:**
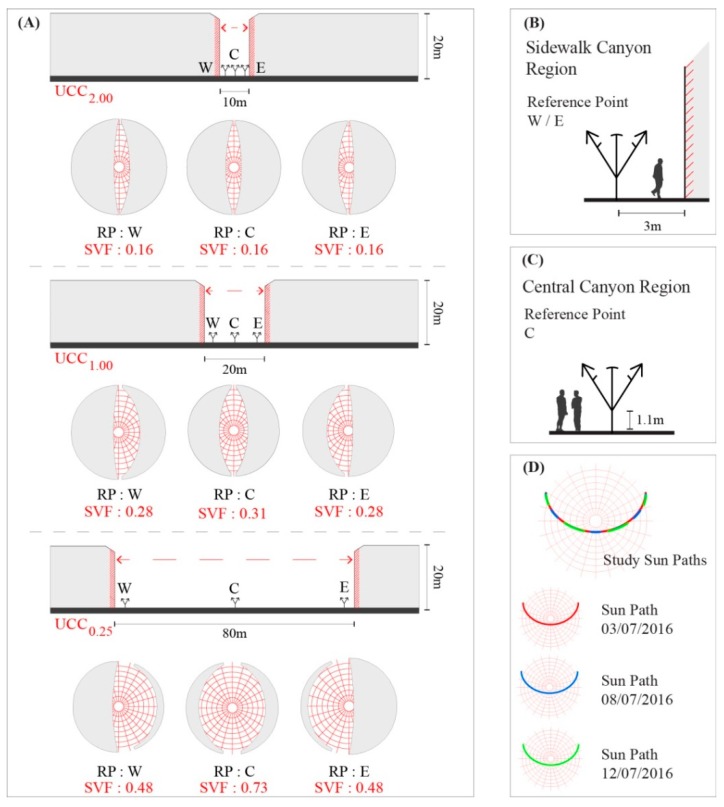
(**A**) The layout of the sky-view-factor (SVF) in each reference point (RP) within the three configured urban canyon cases (UCC). (**B**) Illustration of West or East RP in each UCC. (**C**) Illustration of Central RP in each UCC. (**D**) Illustration of Sun Paths for 3, 8, and 12 July 2016.

At this point in the study, the applied sky-view-factor (SVF) was set equal to 1.00 (or 100%), which correlated to a total absence of urban obstacles or structures. Referring to the earlier study of Lin, et al. [71], the calculation of SVF was based on a classic single-point SVF (or SVF_SP_) within a fixed point, so as to obtain a “fisheye view”, with a calibrated height of 1.1m (which is complacent with the gravity centre of the human body as described by Kuttler [63]). As shown in Figure 1, each fixed point (henceforth reference point (RP)) within the UCCs was tested within the SkyHelios model (Research Center Human Biometeorology; Freiburg, Germany; http://www.urbanclimate.net/skyhelios/) [72,73], to identify the SVF_SP_ at each RP. Such a methodology was already utilized by numerous other studies ([23,54,69] for symmetrical canyons, and [55,74] for asymmetrical canyons). Within this study, and referring to the predominant symmetry that is characteristic of Lisbon’s historical quarter, all of the modelled UCCs were symmetrically constructed. In addition, only the north-to-south orientation was modelled, as this was sufficient to assess how urban structures could lead to different human thermal comfort conditions by evaluating the influence of the geo-referenced sun path upon the morphological composition of each UCC.

As illustrated in Table 2 and Figure 1A, three SVF_SP_ were distributed throughout each canyon, in order to identify the thermo-physiological conditions within their different areas (i.e., Western RP (RP_W_), Central RP (RP_C_), and Eastern RP (RP_E_)). Within the lateral points, RP_W/E_ was adjusted 3 m away from the building façade to typify a pedestrian sidewalk area within the UCCs (Figure 1B). In the case of the central regions, RP_C_ was centered precisely within the middle of the UCCs (Figure 1C).

Through the application of the RayMan and SkyHelios models, Figure 1D represents the geo-referenced sun paths of three selected days (Table 3), these being the following: (1) 3 July, which was identified as a VHD because of the maximum registered T_a_ surpassing 35.0 °C, with generally low *V*_1.1_ and Octas; (2) 8 July, which was identified as a “typical summer day”, with T_a_ averaging at 29.4 °C, in addition to typical RH, *V*_1.1_ and Octas values for the summer period; lastly, (3) 12 July, which represented a “cooler summer day”, where the maximum T_a_ did not reach the ‘typical summer day’ threshold (25.0 °C), and *V*_1.1_ was particularly elevated throughout the day, with a mean of 4.1 m/s. As to be expected, as these three days were not far apart from another, their sun paths did not vary much from one another.

#### 2.3.3. Establishing Synoptic Climate Change Aggravations

In order to construct a synoptic evaluation of how the identified climatological and bioclimatic conditions could be aggravated until the end of the century, the thermo-physiological projections specified by Matzarakis and Amelung [31] were applied. The projections were based on singular climatic variables (T_a_, RH, *V*, and mean monthly sunshine fraction values) obtained from the Climatic Research Unit 1.0 and the HadCM3 datasets. Once obtained, the variables were processed within the RayMan model to obtain the PET and mean radiant temperature (T_mrt_) values for each specific grid. Lastly, this process was also undertaken for the historical period 1961–1990, to establish the respective deviation of PET/T_mrt_ for the end of the century (i.e., CNTRL (or Control) period). The analysis was undertaken by referring to the Special Report of Emission Scenario (SRES) [75] of A1FI, which equated to the fastest and most dramatic effects of global climate change. More recently, this scenario would now equate to the Representative Concentration Pathway (RCP) [76] of 8.5, now comparable to the previous worst-case-scenario of A1FI [77].

Under the examined scenario, Matzarakis and Amelung [31] identified that specifically for the Mediterranean region, the PET values revealed considerable increases (including excesses of 15.0 °C) by the end of the century. These values sharply diverged from the estimated increase of up to 4.0 °C, under the same worst-case-scenario by the Intergovernmental Panel on Climate Change [78]. More concretely for the case of Lisbon, the projected augmentation of PET varied approximately between +10 °C and +12.5 °C. Based on these values and referring to the “what if?” approach discussed/applied by the authors of [22,25,79,80], a synoptic estimation of potential local climatic and bioclimatic territorial impacts on human health and comfort was thus permitted.

Centered on the augmentation of +10 °C in PET by the end of the century to establish a pilot estimation of future thermo-physiological conditions, it was also required to consider the extension of the original PS grades as delineated by Matzarakis and Rutz [57] (Table 1).

As shown in Table 4, three additional ‘Extreme Heat Stress’ grades were added based on an incremental PET increase of 5 °C beyond the value of 41 °C. Naturally, this extension raises the opportunity for future study and refinement, including how the related levels of stress could more concretely strain the human biometeorological system. This being said, the variation and distribution of thermo-physiological indices, and their respective calibration against stress levels have already been launched within numerous studies [14,16,20,22,25,56,81,82].

#### 2.3.4. Identifying Overall Thermo-Physiological Loads and Cumulative Stress

Estimating the average thermal stimuli per hour using different thermal indices has become the standard method to present thermal comfort conditions within a specific place and time, yet, as argued by Charalampopoulos, Tsiros, Chronopoulou-Sereli, and Matzarakis [20], “there is a need, however, to consider the total thermal load caused by the attendance of a person in an open space during a specific time period (…) More specifically, to approach the real effect of the environmental configuration of an open urban space on human health and comfort, the cumulative heat stress caused by each open space configuration should be considered” (p. 1). Guided by their methodology, the PET load (PETL) and the cumulative PETL (cPETL) were applied to the results obtained from the previous sections in this study, whereby (i) PETL refers to the outcome difference from the optimum thermal conditions, permitting the identification of a concrete value, which denotes explicitly the amount of thermal strain on “optimum thermal conditions” (Equation (2)); and, (ii) cPETL accounts for the cumulative sum of the PETL specifically during a predetermined set of hours (Equation (3)). Similarly adapted by Nouri, Costa, and Matzarakis [22], the following equations were utilised for this section:

(2)  PETL=PETh−BC 

where PET*h* is the average hourly PET value, and *BC* (i.e., background conditions) in this section was set to denote the maximum PET for the PS grade of “no thermal stress” (i.e., a PET of 23 °C).

(3) $$\;M\mathrm{cPETL}=\sum_{h=9}^{12}\mathrm{PETL}\;A\mathrm{cPETL}=\sum_{h=13}^{18}\mathrm{PETL}\;D\mathrm{cPETL}=\sum_{h=9}^{18}\mathrm{PETL}\;$$

where *M* ≜ is the morning period (09:00–12:00), *A* ≜ is the afternoon period (13:00–18:00), and *D* ≜ is the diurnal period (09:00–18:00).

Considering the thermal comfort conditions presented within one of the UCCs, PETL was utilized to summarize conditions within a specific urban morphological setting for the three different types of days extracted from the |Jul_2016_| dataset. In addition, and through the “what if?” approach, the assessment was compared against the results in light of the potential increase in PET values in light of the RCP 8.5/SRES A1FI scenario by the end of the century. Lastly, and returning to the thermal comfort conditions processed directly from the meteorological station, cPETL was considered for the entire |Jul_2016_| data set in order to obtain an overall comprehension of both the intensity and periodicity of thermo-physiological stress loads for both existing and projected future conditions.

## 3. Results and Discussion

### 3.1. July Datasets

#### 3.1.1. Singular Variable Heatmaps

When considering the singular climatic results obtained from the meteorological station for the |Jul_2012_|, |Jul_2013_|, |Jul_2014_|, |Jul_2015_|, and |Jul_2016_| datasets, it was possible to identify the following: (i) the overall monthly variance of the different climatic variables for the month of July for 2012 through to 2016; (ii) the diurnal oscillation of variables throughout the month, permitting the identification of climatic events, including HWE and VHD; and (iii) the hourly oscillation of the variables between the hours of 09:00 and 18:00.

**Figure 2:**
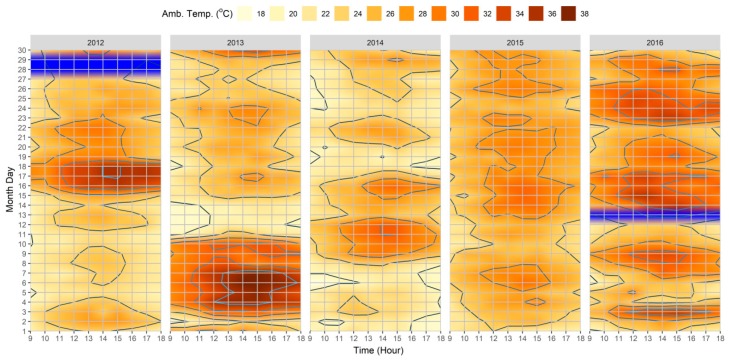
Heatmap of hourly variations of air temperature (T_a_) between 09:00–18:00 for 2012–2016 extracted from Lisbon’s meteorological station (N°08535). The blue color corresponds to the station calibration (CAL.) periods.

As shown in Figure 2, when reviewing the T_a_ results, it was possible to identify substantial differences between the datasets. Comparatively between the five, |Jul_2014_| and |Jul_2015_| were the datasets with generally lower T_a_ values. On the other hand, |Jul_2013_| and |Jul_2016_| were the datasets with the highest values, with frequent extreme climate events, including an eight-day HWE, which was also accompanied by a sequential four VHD in |Jul_2013_|. In the case of |Jul_2016_|, although shorter, three separate HWE were identified throughout the month, accompanied by four VHD. Moreover, as the values were recorded hourly, it was possible also to ascertain that even within the “cooler” datasets, the hours between 12:00 and 15:00 still witnessed elevated T_a_ values, namely during the short HWE during 11 and 12 July in the |Jul_2014_| dataset.

**Figure 3:**
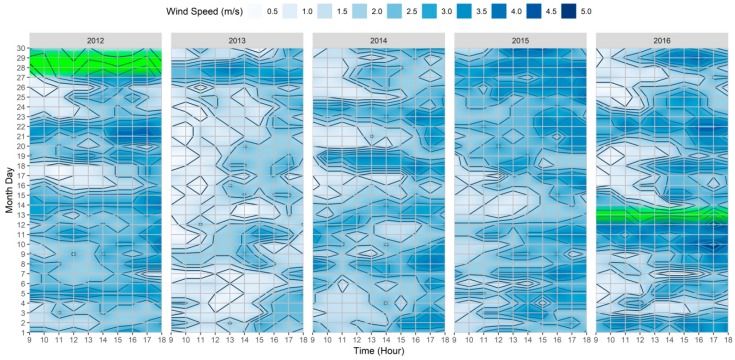
Heatmap of hourly variations of *V*_1.1_ between 09:00–18:00 for 2012–2016 extracted from Lisbon’s meteorological station (N°08535). The green color corresponds to the station CAL. periods.

Unlike the T_a_ results, and as shown in Figure 3, *V*_1.1_ tended to indicate stronger hourly oscillations because of its intrinsic higher rate of fluctuation [83]. That being said, it was possible to identify general trends amongst the datasets. Particularly in the case of |Jul_2013_| and |Jul_2014_|, where diurnal *V*_1.1_ was generally lower, the morning period tended to reveal lower speeds in comparison to those registered for the later part of afternoon period. With regards to higher *V*_1.1_ values, both the beginning of the |Jul_2012_| dataset and end of the |Jul_2015_| dataset presented the longest periods of higher speeds. Nevertheless, it was identified amongst the datasets that there were also short periods (i.e., frequently between two and three days), which revealed higher *V*_1.1_ values. To a certain degree, during these short periods, it was possible to verify the corresponding decreases in T_a_ as exemplified by the following: (i) 17th–19th and 23rd–24th in the |Jul_2014_| dataset; and (ii) 1st–2nd, 10th–12th, and the 21st–22nd in the |Jul_2012_| dataset. On the other hand, the days/hours with particularly high T_a_ values often presented lower *V*_1.1_ values as exemplified by the following: (i) 16th–18th in the |Jul_2012_| dataset; (ii) 30th–31st in the |Jul_2013_| dataset; and (iii) 13th–15th in the |Jul_2015_| dataset. It was noted however that the correlation between the two variables did not always take place, as exemplified by higher *V*_1.1_ values during the afternoon period during the identified VHD/HWE in the |Jul_2013_| dataset.

**Figure 4:**
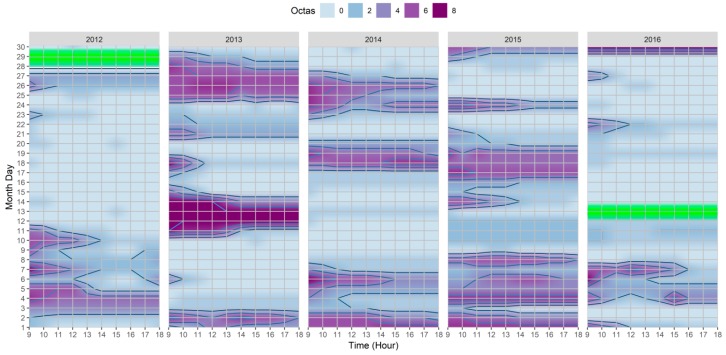
Heatmap of hourly variations of Octas between 09:00–18:00 for 2012–2016 extracted from Lisbon’s meteorological station (N°08535). The green color corresponds to the station CAL. periods.

**Figure 5:**
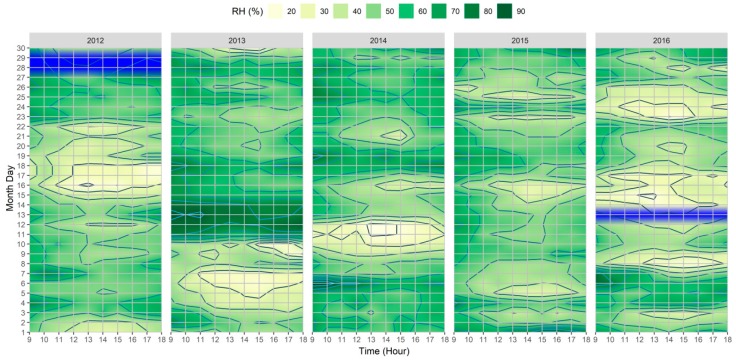
Heatmap of hourly variations of relative humidity (RH) between 09:00–18:00 for 2012–2016 extracted from Lisbon’s meteorological station (N°08535). The blue color corresponds to the station CAL. periods.

When considering the outcomes shown in Figure 4 and Figure 5, it was possible to identify correlations between Octas and RH, and moreover, with the other variables. In general, when higher Octas values were registered by the meteorological station, the RH tended to be correspondingly higher, as exemplified by the following: (i) 3rd–4th in the |Jul_2012_| dataset; (ii) 11th–14th in the |Jul_2013_| dataset; (iii) 6th/18th/31st in the |Jul_2014_| dataset; (iv) 1st/4th/9th/24th in the |Jul_2015_| dataset; and (iv) 4th–7th in the |Jul_2016_| dataset. Furthermore, and considering relationships with other variables such as T_a_, and as exemplified by the VHD/HWE in the |Jul_2013_| dataset, the higher air temperature was complemented with the days with low Octas/RH. Similarly, and still within the same dataset, just after these days with a very high air temperature a drop in T_a_ between the 11th–14th of the month was accompanied by increases of both Octas and RH; correspondingly, and although for shorter periods, similar correlations were also particularly discernable throughout the |Jul_2016_| dataset.

#### 3.1.2. Thermo-Physiological Heatmaps

Often within the previous section, it was possible to identify correlations between the singular variables within the different datasets. However, it was also identified that these correlations were not always clear, and usually took place when a specific variable was particularly high, including high T_a_ values during VHD/HWE. As a result, when approaching the human thermal comfort thresholds, a single unit that considers the interactions of all of the singular variables becomes essential. Consequently, the thermo-physiological indices of PET and mPET were processed in this section so as to obtain a wholesome understanding of how the amalgamation of variables could influence the human thermal comfort thresholds.

**Figure 6:**
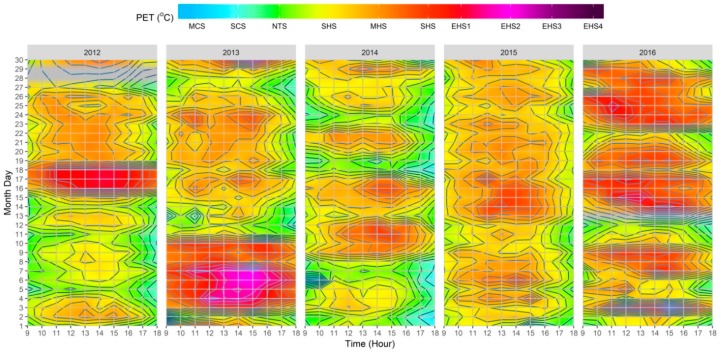
Heatmap of hourly variations of physiological stress (PS) grades based on physiologically equivalent temperature (PET) values between 09:00–18:00 for 2012–2016, based on the fusion of singular climatic variables extracted from Lisbon’s meteorological station (N°08535). The grey color corresponds to the station CAL. Periods.

As presented in Figure 6, the PET results illustrate how a wholesome understanding can be obtained through the combination of all of the variables through the use of the RayMan model. As already stated, the reason for using PET was because of its calibration being dependent on easily accessible data, and moreover, it’s measuring unit being based on °C. As demonstrated throughout the datasets, the utilization of PET permitted a more straightforward assessment of thermal comfort conditions. In addition, and by reviewing the PS grades as presented in Table 1 and Table 4, it was possible to determine the hourly thermal stress grades on the human biometeorological system.

Between all of the individual climatic variables, the T_a_ results presented the most significant similarity to the thermo-physiological indices. Nevertheless, as the influence of other variables was not reflected, T_a_ alone was far less efficient to provide a complete evaluation of human thermal comfort conditions, as exemplified by the following: (1) continuation of higher thermal stress when T_a_ values decreased after the VHD/HWE in the |Jul_2012_| dataset, likely attributable to the permanency of low Octas values until the 26th; (2) high PET values during the VHD/HWE in the |Jul_2013_| dataset, which beyond high T_a_ values, can be also attributed to low *V*_1.1_, Octas, and RH values between the 3rd–8th; (3) the lower PET values between the 11th–14th in the |Jul_2013_| dataset, which beyond lower T_a_ values, can also be interlinked to particularly high Octas and RH values for those days; (4) occurrence of some mild cold stress (i.e., PET < 22 °C) in the morning and late afternoon as demonstrated within the |Jul_2014_| dataset which can be associated to lower T_a_ and higher RH and *V*_1.1_ values; and (5) resulting influences of days with both lower T_a_ and higher *V*_1.1_ values on thermal comfort conditions which were particularly salient within the |Jul_2016_| dataset.

**Figure 7:**
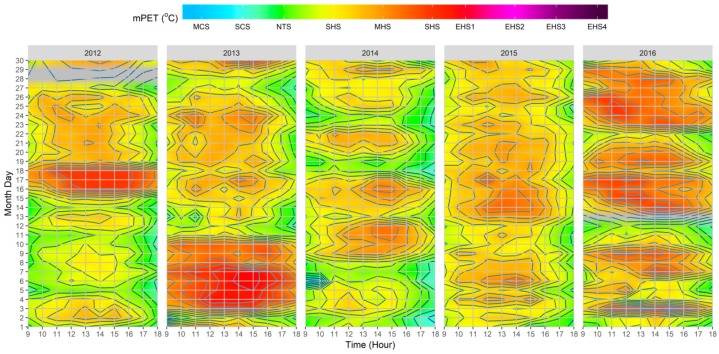
Heatmap of hourly variations of physiological stress (PS) grades based on modified physiologically equivalent temperature (mPET) values between 09:00–18:00 for 2012–2016, based on the fusion of singular climatic variables extracted from Lisbon’s meteorological station (N°08535). The grey color corresponds to the station CAL. periods.

When considering the results presented in Figure 7, it was possible to identify that the adapted index presented similar outcomes to those presented by (i) the original study conducted by Chen and Matzarakis [60] for the city of Freiburg; (ii) the study undertaken by Lin, Yang, Chen, and Matzarakis [62] for the hot and humid conditions in Taiwan; and, (iii) the study elaborated by Nouri, Costa, and Matzarakis [22], which was also undertaken for the downtown district of Lisbon. More specifically, and particularly during the periods of higher thermal stimuli on the human body, it was verified that mPET revealed no periods that surpassed the “extreme heat stress” grade. The identified difference was particularly noteworthy between 12:00–16:00 on the 4th–7th within the |Jul_2013_| dataset, where the maximum presented PS grade was of “extreme heat stress”, unlike PET, which reached the second level of “extreme heat stress” for the same period. In addition, it was also possible to determine that the mPET index also revealed a higher tendency to present values within the “No thermal stress” grade, both in the circumstances of cold stress (e.g., during the morning and late afternoon of the |Jul_2013_| and |Jul_2014_| datasets) and heat stress (e.g., during the afternoon in all datasets).

### 3.2. Urban Canyon Case Datasets

So far, within the existing literature, the crucial role of urban morphology (i.e., the width, height, and orientation of urban canyons) on thermal comfort conditions within the built environment has been well documented [22,23,54,55,69,70,74,84,85]. Therefore, and within this section, the UCCs were utilized to identify how different morphological compositions present different thermal comfort conditions from those presented by the meteorological station during three different types of days retrieved from the |Jul_2016_| dataset. Analogously, both the PET and mPET results were also presented for comparative purposes within the three RPs for each UCC.

#### 3.2.1. “Cooler Summer Day”—12 July

**Figure 8:**
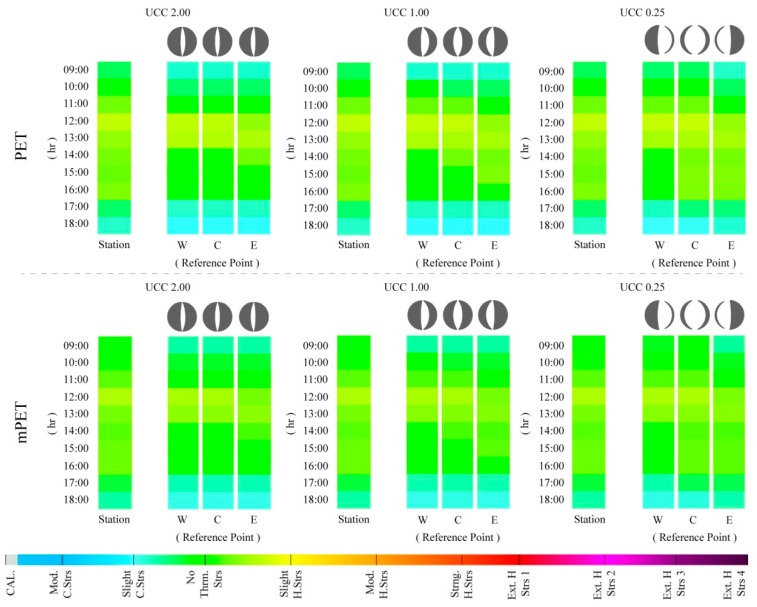
Hourly variations of physiological stress (PS) grades based on physiologically equivalent temperature (PET) and modified physiologically equivalent temperature (mPET) values between 09:00–18:00 for 12 July 2016 within the urban canyons cases (UCCs).

When considering the results for 12 July, which was a comparatively cooler day due to the comparatively elevated diurnal *V*_1.1_ (with a mean of 4.1 m/s) and lower T_a_ (with a mean of 23.2 °C) (Table 3), it was possible to identify that the PS levels ranged predominantly between “slight cold stress” and “slight heat stress” (Figure 8). As expected, between the three UCCs, the case that presented the lowest amount of PS was UCC_2.00_, because of its lower canyon width that reduced its exposure to radiation fluxes. On the other hand, UCC_0.25_ revealed slightly higher PS levels as a result of its higher susceptibility to radiation fluxes, given its higher SVF_SP_, which ranged between 0.48 and 0.78 (Figure 1) for the lateral and central RPs. Furthermore, it was also noted that in each UCCs, all of the three RPs within the canyons presented different PS grades to those presented by the meteorological station, particularly at 09:00 and 17:00–18:00, where PS were slightly lower than those identified by the station. In contrast, and with an exception of RP_E_, all of the other RPs within the three UCCs presented either a continuation or amplification of PS grades obtained by the meteorological station at 12:00.

When comparing the mPET results for 12 July, it was identified that the modified index did not present significant divergences from PET. Nonetheless, it was again possible to verify that the mPET index tended to remain closer to the “no thermal stress” grade, particularly when the PET index tended to either oscillate to either “slight cold stress” or “slight heat stress”. 

#### 3.2.2. “Typical Summer Day”—8 July

**Figure 9:**
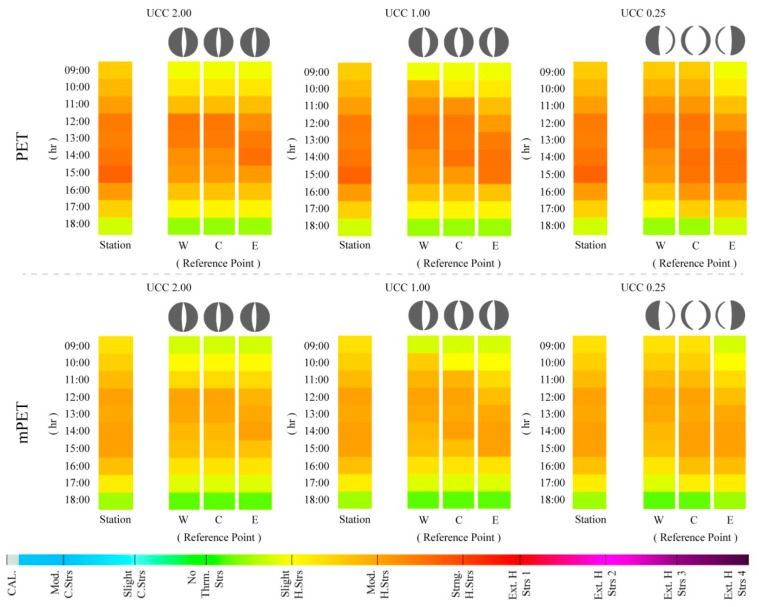
Hourly variations of physiological stress (PS) grades based on physiologically equivalent temperature (PET) and modified physiologically equivalent temperature (mPET) values between 09:00–18:00 for 8 July 2016 within the urban canyons cases (UCCs).

Centered on the classification of a “typical summer day”, the results for 8 July presented fairly different results to those obtained for 12 July. As demonstrated in Figure 9, it was possible to identify that in the case of PET, the PS levels frequently reached “strong heat stress” during the afternoon. Furthermore, unlike the previous assessment, 12 July led to greater disparities between the PS results presented by the meteorological station and those within the UCCs. The divergence was particularly perceptible within UCC_2.00_ and UCC_1.00_ during the morning period (i.e., between 09:00 and 11:00), where PS tended to vary an entire grade (equating to PET variance of ~6.0 °C). Generally, the hours between 12:00 and 15:00 were the hours with the highest PS grades, which, in the case of the PET index, reached “strong heat stress”. Conversely, in the case of UCC_0.25_RP_W_ and UCC_0.25_RP_C_, the PS grades were more similar with those presented by the meteorological station, where the morning stress grades were closer to the “moderate heat stress” grade. The reason for this can be attributed to the increased width of the UCC_0.25_, thus presenting a greater vulnerability to radiation fluxes, with the exception of UCC_0.25_RP_E_, which was cast in the shade during the morning period. It was additionally identified that, similar to 12 July, the PS grades within the UCCs were almost always lower than those presented by the meteorological station. This variance ultimately relays to the attenuating influence of urban morphology on local human thermal comfort conditions.

With regards to the mPET index, a greater variation from the PET results was manifested, especially during the afternoon, where PET reached a PS grade of “strong heat stress”. During this period, mPET frequently revealed a PS of “moderate heat stress”. At 09:00, mPET also revealed PS grades that were closer to “no thermal stress” in comparison to those revealed by PET.

#### 3.2.3. “Very Hot Summer Day” (VHD)—3 July

**Figure 10:**
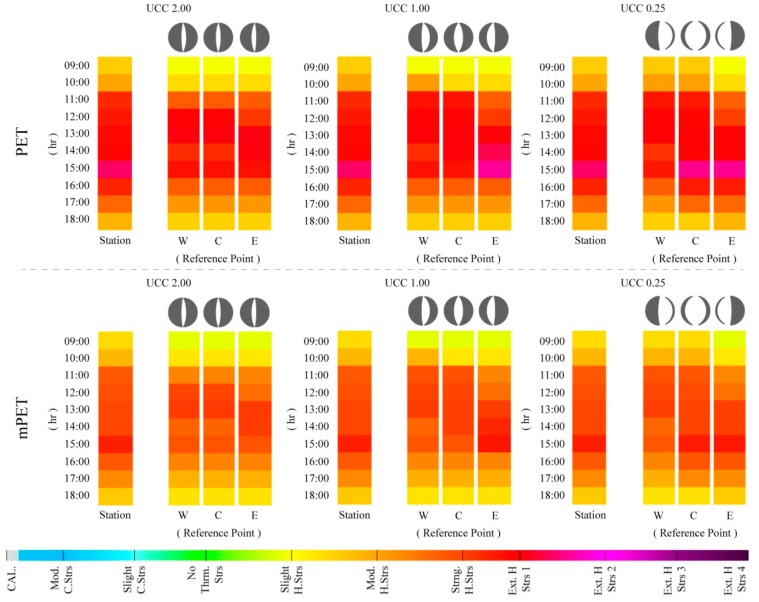
Hourly variations of physiological stress (PS) grades based on physiologically equivalent temperature (PET) and modified physiologically equivalent temperature (mPET) values between 09:00–18:00 for 3 July 2016 within the urban canyons cases (UCCs).

In the case of the hottest day (i.e., a VHD) within the |Jul_2013_| dataset, 3 July revealed the strongest variations between the results obtained for the UCCs, and those from the meteorological station (Figure 10). In addition, it was the first circumstance in which the UCCs presented a higher PS grade to those presented by the station, as exemplified at 15:00 in UCC_1.00_RP_E_, UCC_0.25_RP_C_, and UCC_0.25_RP_E_. These variations did not only take place within wider UCCs, where canyon widths permitted the RPs to obtain higher SVF_SP_ values, as shown in Figure 1. Similar divergences were also observed in UCC_2.00_RP_W_ and UCC_2.00_RP_C_ at 12:00, where the PS grades surpassed the first grade of “extreme heat stress”. During 12:00 and 13:00, both of these locations were exposed to the sun before being cast in the shade at 14:00, which led to a slight reduction of PS. At 15:00, and although still cast in the shade, climatic conditions (RH = 32.6%, *V*_1.1_ = 1.0 m/s, Octas = 0.00, and T_a_ = 35.9 °C) led the PS grades to increase once more. Although also illustrated on 8 July, the results for 3 July presented larger alterations in the PS grades between the different RPs in each UCC. More specifically, and between 11:00 and 15:00, the PS grades frequently varied by up to one entire grade, which can be attributed to the variation of radiation exposure within the assessed canyons. As the processed climatic variables were identical for the UCCs, the RPs and meteorological station, the influence of non-temperature variables such as radiation fluxes proved to be a critical factor for assessing the in situ human thermal comfort conditions.

In comparison with the two previous days, the mPET values also led to higher PS grades. However, these grades were still significantly lower than those presented by the PET index. Even at 15:00, and as exemplified by UCC_1.00_RP_E_, UCC_0.25_RP_C_, and UCC_0.25_RP_E_, mPET did not surpass the first grade of “extreme heat stress”. This drop of PS grades obtained by the mPET index was also generally evident between 11:00 and 15:00 for all of the UCCs.

### 3.3. Synoptic Projections of Thermal Conditions for the Urban Canyon Cases by 2100

#### “What if?” Projections Extrapolated from the 8 and 3 July Outputs

Constructed on the utilization of the exploratory “what if?” approach, the values obtained for 8 and 3 July were modified to synoptically consider how existing human thermal comfort conditions could be theoretically aggravated as a result of potential climate change impacts by the end of the century. As discussed in the methods section, the projections identified by Matzarakis and Amelung [31] were utilized to consider a PET increase of +10 °C on the current values given the occurrence of a global RCP8.5/SRES A1FI scenario. Consequently, as further study is required to consider these augmentations on the mPET index, only the PET values were utilized for the final sections of the study.

**Figure 11:**
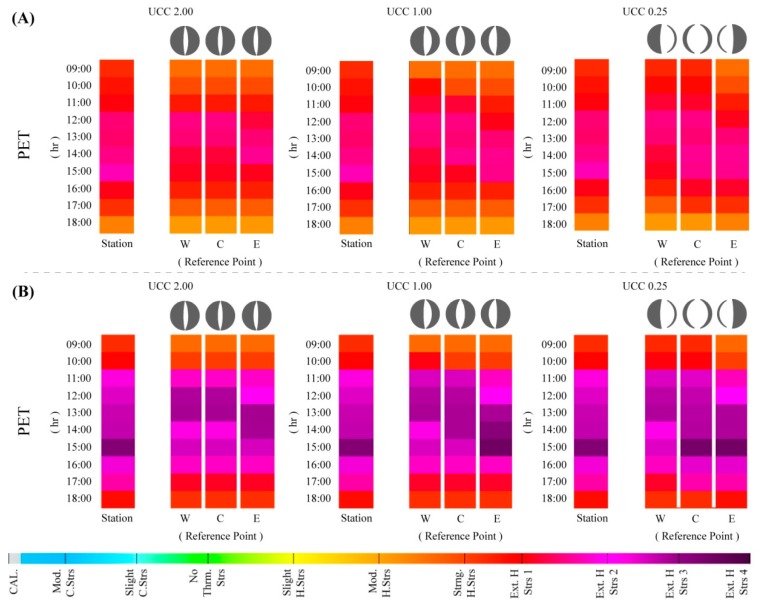
Hourly variations of physiological stress (PS) grades based on physiologically equivalent temperature (PET) and modified physiologically equivalent temperature (mPET) values between 09:00–18:00, based on 8 (**A**) and 3 (**B**) July 2016, with projected climate change augmentations (RCP8.5/SRES A1FI scenario) within the urban canyons cases (UCCs).

As illustrated within Figure 11A and given a worst-case-scenario of climate change until the end of the century, local human thermal conditions presented very different values to those observed for 8 July, representing a “typical summer day”. Although the morning period continued to present lower PS conditions to those presented by the meteorological station, it could not be overlooked that as early as 09:00, the PS values were already at “strong heat stress” and “extreme heat stress” within UCC_0.25_RP_W_ and UCC_0.25_RP_C_. Successively, and for most of the afternoon period, while the UCC values were lower, the PS values still ranged from the first and second level of “extreme heat stress”. These results indicate that while the existing human thermal results are already alarming, they can be considerably aggravated given a typical hot dry Mediterranean summer. Furthermore, it was additionally acknowledged that the heightened thermal stimuli took place in all UCCs, and moreover, within all of their respective RPs.

Within Figure 11B the results from the estimated aggravation of thermo-physiological conditions obtained for 3 July (i.e., a VHD) are presented. In comparison to the previous synoptic projections that were grounded on a “typical summer day”, the results based on a VHD presented dramatic thermal stress levels, reaching a maximum PET value of 58.3 °C in UCC_0.25_RP_E_, and 58.3 °C in UCC_1.00_RP_E_ at 15:00. These PET outcomes resulted in PS levels ranging up to the fourth level of “extreme heat stress”, thus raising alarming implications for human thermal comfort conditions. Comparatively between the different UCCs, only the UCC_2.00_ did not reach the utmost level of “extreme heat stress”, nevertheless in all of the RPs; there was still a two-hour period in which the PS levels reached the third level of “extreme heat stress”. The obtained results imply that within the worst-case-scenarios associated to climate change by the end of the century, regardless of urban morphology, human thermal comfort conditions within the built environment can potentially reach very high levels of thermo-physiological strain. 

### 3.4. Present and Future Thermo-Physiological Loads and Cumulative Stress

#### 3.4.1. The Intensity of Thermo-Physiological Loads beyond Comfortable Conditions

**Figure 12:**
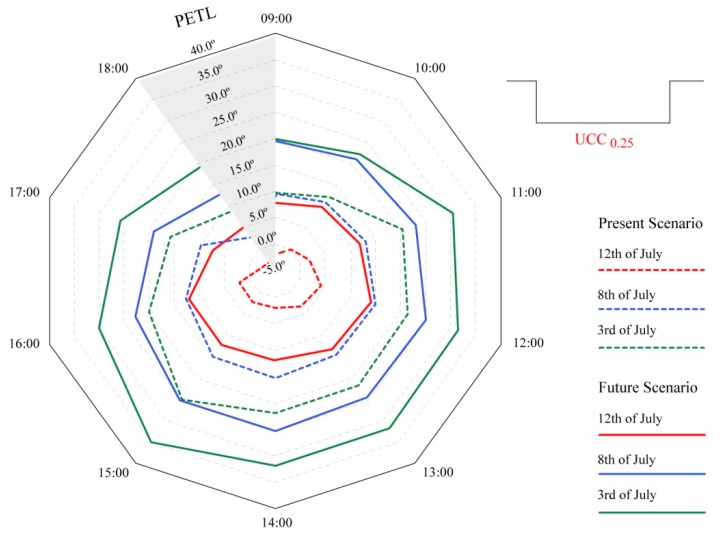
Radar chart of distribution for existing and projected diurnal physiologically equivalent temperature load (PETL) values within urban canyons case 0.25 (UCC_0.25_) for 3, 8, and 12 July.

Established on a value that was representative of “optimum thermal conditions”, the thermal load on the human body could be established through the adapted PET index, PETL. This assessment was carried out for the UCC_0.25_RP_C_ because of its greater susceptibility to higher thermal stimuli. As presented in Figure 12, based on considering any PET value above 23 °C as an additional thermal load beyond the grade of “no thermal stress” (or BC as presented within Equation (2)), it was possible to ascertain estimates of thermal stress loads for both existing, and projected future conditions. 

As already discussed, out of the three days used for the UCC investigations, 12 July presented the lowest PS grades because of a combination of both lower diurnal mean T_a_ values and higher *V*_1.1_ speeds. For this reason, and during the morning period, PETL revealed values below 0.0 °C, namely at 09:00 and 18:00, where PETL values reached −2.1 °C and −4.9 °C, respectively. Both in the case of the hotter days in July, the PETL values varied between: (i) 2.9 °C (at 18:00) and 15.3 °C (at 15:00) for 8 July; and (ii) 19.5 °C (at 09:00) and 35.1 °C (at 15:00) for 3 July. These values once again indicate that the existing conditions already present considerable thermal stimulus on the human body; particularly at 15:00, which in accordance with results in the previous section, was revealed to be the period with the highest amount of thermal stimulus. For this reason, and given that Mediterranean climates with hot and dry summers already present significant thermal “risk factors” during VHD/HWE events, approaches to attenuating thermal comfort conditions at local scales already play an imperative role for the welfare of outdoor comfort in consolidated urban environments.

When considering future bioclimatic conditions within the UCCs, and analogous to the outcomes presented in Figure 11, the results of Figure 12 reveal how potential climate change impacts could lead to dramatic increases of urban thermo-physiological stress loads on pedestrians. In the case of 12 July, its hourly PETL values were comparable to those presented by an existing “typical summer day”. This similarity theoretically inferred that the thermal loads expected during a future “cooler summer day” could be concomitant with those found within what is now considered a “typical summer day”. When considering the results for 8 July within a future scenario, hourly PETL intensities increased dramatically, whereby between 11:00 and 15:00, the PETL values ranged from 23.0 °C to 25.3 °C. In terms of thermal comfort thresholds, these results imply a drastic divergence from “no thermal stress” conditions for a future “typical summer day”, where, moreover, the PETL values frequently surpassed those obtained for an existing VHD. Lastly, and in the case of the projected thermal effects obtained for 3 July, the PETL values were severe for the majority of the day, with values constantly remaining between that of 30.4 °C and 35.1 °C from 11:00 until 16:00. For this reason, while existing extreme events such as VHD already present alarming conditions for human health and comfort, the potential aggravating effects associated to the climate change RCP8.5/SRES A1FI scenarios can possibly reveal far more extreme thermo-physiological stress. 

#### 3.4.2. The Periodicity of Cumulative Thermo-Physiological Stress Load

Adjacently to the intensity of thermo-physiological stress, considering that the periodicity of the thermal stimuli is just as significant, as the exposure to outdoor conditions can last for various hours. As a result, and as suggested by the work elaborated by Charalampopoulos, Tsiros, Chronopoulou-Sereli, and Matzarakis [20], cumulative assessments are also required to obtain an estimation of the thermal stress resultant of remaining under set conditions for a given amount of time. As specified by Equation (3), within the methodology section, the values for *M*cPETL, *A*cPETL, and *D*cPETL from the |Jul_2016_| dataset were utilized to obtain an overall comprehension of cumulative thermal stress for each of the 31 days of the month.

**Figure 13:**
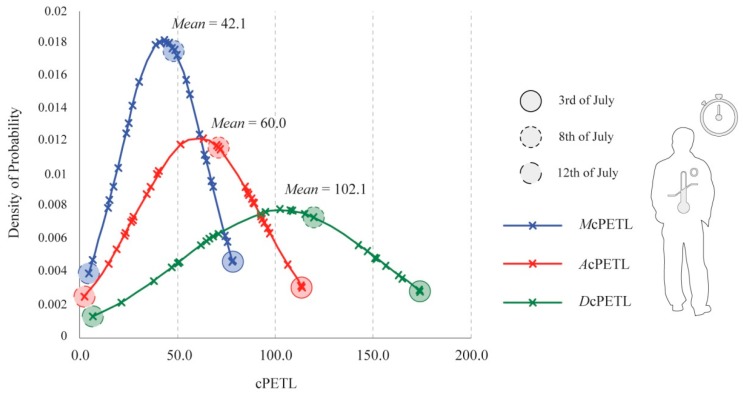
Bell curve comparison for existing morning, afternoon, and diurnal cumulative physiologically equivalent temperature loads (cPETL) for July 2016.

**Figure 14:**
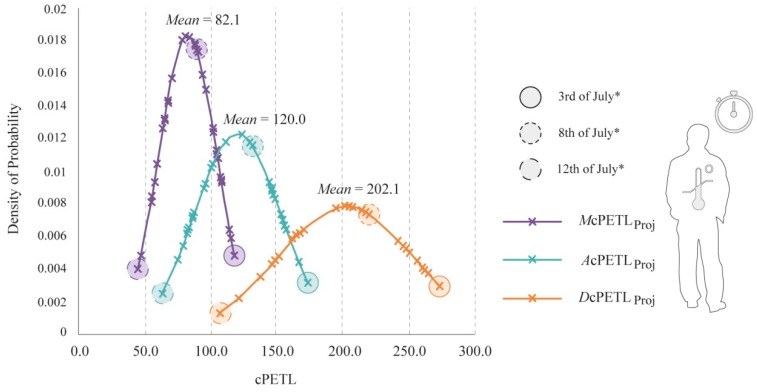
Bell curve comparison for projected morning, afternoon, and diurnal cumulative physiologically equivalent temperature loads (cPETL) for July 2016, with projected climate change aggravations * (RCP8.5/SRES A1FI scenario).

As revealed in Figure 13, *M*cPETL, *A*cPETL, and *D*cPETL presented different bell curves resultant of the different susceptibilities to cumulative thermal stress. *M*cPETL revealed that the morning period presented the lowest quantity of cumulative stress load; whereby if a person were to remain outdoors for the morning period between the hours of 09:00 and 12:00 during July 2016, they would be susceptible to an estimated mean cPETL of 42.1. On the other hand, in the case of *A*cPETL, the afternoon period revealed a higher mean cPETL of 60.0, because of the exposure of hours that frequently presented the highest thermal stimuli. In the case of *D*cPETL, which equated to the combination of both the morning and afternoon periods, a person that remained outdoors for the entire day would be exposed to an estimated mean cPETL of 102.1. Amongst the thirty-one days of July, the location 3, 8, and 12 July were highlighted within the bell curve to demonstrate their particular rate of probability (Figure 13 and Figure 14). Naturally, due to the notably colder/hotter conditions during both 12 and 3 July, these days were situated at opposite ends of the bell curve, implying considerably divergent amounts of cPETL within all of the stipulated timeframes. In contrast, 8 July, representing a “typical summer day”, had the highest probability of the three days, with cPETL values not oscillating far from obtained mean values.

In comparison to the cPETL values presented for the existing thermal conditions, potential future augmentations of thermo-physiological stress led to considerably different bell curves, as revealed in Figure 14. Generally, there was almost a two-fold-increase in the cPETL values for all of the temporal periods, leading to significantly higher mean values for *M*cPETL_Proj_, *A*cPETL_Proj_, and *D*cPETL_Proj_. Moreover, even in the case of *M*cPETL_Proj_ during the corresponding coolest day of the month, cPETL was of 44.2, which was higher than the mean value for the existing *M*cPETL. Similarly, for *A*cPETL_Proj_, the coolest day presented a cPETL of 62.3, which surpassed the mean value for an existing “typical summer day”. Respectively, the modified estimated mean for *A*cPETL_Proj_ severely amplified to 120.0, which was significantly higher than the acquired mean of 102.1 for the *D*cPETL presented in Figure 13. With regards to *D*cPETL_Proj_, all of the cPETL values were very high, whereby its (i) mean cPETL was well beyond what would be expected during a current VHD; (ii) the lowest cPETL was of 106.5, which surpassed the mean value obtained for *D*cPETL; and lastly, (iii) the highest estimated cPETL ranged up to 274.8, a value that invariably implied the reduced feasibility of long-term pedestrian permanency in outdoor contexts under the disclosed climate change scenario. 

Such outcomes again refer to the two interrelated rationales that exist within Mediterranean climates with hot and dry summers, namely: (1) existing thermal comfort thresholds are already prone to significant thermo-physiological stress intensity and periodicity during the hotter months of the year; and, in addition, that (2) vulnerabilities to such stimuli can dramatically increase by the end of the century in light of worst-case-scenarios associated to climate change within consolidated urban environments. 

## 4. Concluding Remarks for Human Health and Thermal Comfort

With the objective of approaching human thermal comfort in climates with hot-dry Mediterranean summers, this article examined the following: (i) relationship between singular climatic variables and thermo-physiological indices; (ii) impact of selected urban morphological compositions upon local thermo-physiological indices to identify the crucial role of radiation fluxes; (iii) synoptic aggravations of current local human thermal risk factors, given the occurrence of a potential worst-case-scenario of climate change; and (iv) intensity and periodicity between current conditions and those projected for the end of the century.

- To a certain degree, it was possible to identify correlational “cause-and-effect” relationships between the individual variables, especially during the periods of higher climatic stimuli. However, and as directly revealed by the constructed heatmaps, these relationships were not always straightforward, nor did they provide an overall reflection of human thermal comfort conditions. This result was also pertinent to T_a_ as well. Although this specific variable presented the highest similarity to the thermo-physiological results, as the influences of other variables upon the human body were not reflected, T_a_ was insufficient to present a wholesome evaluation of thermal comfort conditions.
- In continuation from the previous point, the exercise of transposing meteorological station data into urban canyon cases further confirmed these results within Lisbon’s historical district. More specifically, by identifying the central and lateral sky-view-factors, it was possible to undertake precise estimations of global radiation, which rendered clear differences in in situ thermal comfort thresholds. As a result, although the introduced individual variables (including T_a_) retrieved from the station remained constant across the stipulated reference points, the thermo-physiological variables varied drastically (with PET variations of up to ~6.0 °C within the different regions of the canyons).
- Currently, human health and thermal comfort are already prone to extreme physiological stress levels during one of the hottest months of the year in Lisbon. Nevertheless, current extremes could potentially be alarmingly surpassed by the end of the century. In the case of synoptically estimating climate change aggravations on a current “typical summer day”, even in the morning, the projected physiological stress already ranged between “strong heat stress” and “extreme heat stress”. During the afternoon period, the physiological stress values ranged between the first and second level of “extreme heat stress” (representing PET values between 41 °C and 51 °C). Likewise, when considering the aggravations for a current ‘very hot day’, the projected PET values reached a maximum of 58.5 °C.
- As identified within the study, while the existing conditions extracted from the July 2016 dataset already presented high vulnerability rates during the morning, afternoon, and overall diurnal period—the synoptic projected cumulative thermo-physiological stress load values revealed drastic increases by almost 100%. As a result, this indicated that, regardless of the thermal risk factors already existing in Lisbon during the summer period, these conditions could drastically deteriorate. This deterioration would moreover lead to acute impacts upon urban mortality rates, outdoor activity threads, and overall urban well-being by the end of the century.

While it is inarguable that disseminations from entities such as the Intergovernmental Panel on Climate Change have been indispensable for the maturing climate change agenda, when approaching human thermal conditions at local scales, the use of simpler indexes that do not consider critical non-temperature characteristics (e.g., radiation fluxes patterns) shall always likely prove insufficient for concrete local adaptation efforts. For this reason, the use of climate models (e.g., RayMan and SkyHelios) play a fundamental role in approaching local human thermal comfort, because of their capacity to further specify the influences of local morphological characteristics within consolidated urban contexts. Within this study, and focused upon a bottom-up perspective, various methodologies were combined to demonstrate the relationship between singular climatic variables and that of thermo-physiological indices. This assessment was moreover undertaken to demonstrate how both existing and future human thermal conditions could be approached by non-climatic experts (including urban planners, urban designers, and landscape architects) to undertake adaptation initiatives in an era susceptible to further climatic aggravations and uncertainty.

## Figures and Tables

**Table 1:** Ranges of the physiologically equivalent temperature (PET) for different grades of thermal perception and physiological stress (PS) on human beings; internal heat production: 80 W, heat transfer resistance of the clothing: 0.9 clo, according to the authors of [10] (source: adapted from the authors of [61]).

PET	Thermal Perception	Physiological Stress
<4 °C	Very Cold	Extreme Cold
4~8	Cold	Strong Cold
8~13	Cool	Moderate Cold
13~18	Slightly Cool	Slight Cold
18~23	Comfortable	No Thermal Stress
23~29	Slightly Warm	Slight Heat
29~35	Warm	Moderate Heat
35~41	Hot	Strong Heat
>41	Very Hot	Extreme Heat

**Table 2:** Representation and description of utilized default canyons and their respective height (H), width (W), and length (L). UCC—urban canyon cases.

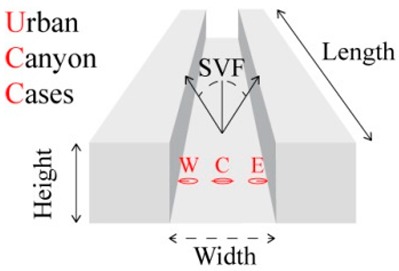	**H/W Ratio**	**Canyon Height (m)**	**Canyon Width (m)**	**Canyon Length (m)**	**Description (UCC)**
	2.00	20	10	200	UCC_2.00_
	1.00	20	20	200	UCC_1.00_
	0.25	20	80	200	UCC_0.25_

**Table 3:** Maximum, mean, and minimum values for singular climatic variables retrieved from Lisbon’s meteorological station (N°08535) for 3, 8, and 12 July 2016. RH—relative humidity.

Day	T_a_			RH			*V*_1.1_			Total Clo. Cover		
	Max.	Mean	Min.	Max.	Mean	Min.	Max.	Mean	Min.	Max.	Mean	Min.
	(°C)			(%)			(m/s)			(Octas)		
3 July 2016	35.9	32.0	26.4	58.6	41.8	32.6	2.6	1.7	1.6	0.0	0.0	0.0
8 July 2016	32.1	29.4	25.9	41.7	29.3	21.4	3.6	2.6	1.6	1.0	0.4	0.0
12 July 2016	24.5	23.2	24.5	50.8	42.0	37.0	5.2	4.1	3.1	1.0	1.0	1.0

**Table 4:** Applied grade extension of physiological stress (PS) on human beings to accompany increased physiologically equivalent temperature (PET) values beyond 41 °C for RCP 8.5/SRES A1FI scenario until the end of the century (See Table 1). Adapted from the authors of [61]. *—additional grade.

PET	Physiological Stress	PS Acronym
…	…	-
8~13	Moderate Cold	MCS
13~18	Slight Cold	SCS
18~23	No Thermal Stress	NTS
23~29	Slight Heat	SHS
29~35	Moderate Heat	MHS
35~41	Strong Heat	SHS
41~46	Extreme Heat Lv.1	EHS1
46~51	Extreme Heat Lv.2 *	EHS2
51~56	Extreme Heat Lv.3 *	EHS3
56~61	Extreme Heat Lv.4 *	EHS4

## Nomenclature

‘*Csa*’	hot-mediterranean climate (-)
*A*cPETL	afternoon cumulative physiologically equivalent temperature load (-)
BC	background conditions
CAL.	calibration period (-)
cPETL	cumulative physiologically equivalent temperature load (-)
CTIS	climate tourism/transfer information scheme (-)
*D*cPETL	diurnal cumulative physiologically equivalent temperature load (-)
H/W	height-to-width (-)
HWE	heat wave events (-)
KG	Köppen Geiger (-)
*M*cPETL	morning cumulative physiologically equivalent temperature load (-)
MEMI	Munich energy-balance model for individuals (-)
mPET	modified physiologically equivalent temperature (°C)
PET	physiologically equivalent temperature (°C)
PETL	physiologically equivalent temperature load (°C)
PS	physiological stress (-) *
RH	relative humidity (%)
RP*_X_*	*X* reference point (-) (W—Western, C—Central, E—Eastern)
SVF	sky-view-factor (-)
SVF_SP_	single point sky-view-factor (-)
T_a_	air temperature (°C)
T_mrt_	mean radiant temperature (°C)
UCCs	urban canyon case studies (-)
UCC*_X_*	*X* H/W Urban Canyon Case (-)
UCC*_X_*RP*_X_*	*X* reference point in *X* urban canyon case (-)
UHI	urban heat island (-)
*V*	wind speed (m/s)
*V*_1.1_	wind speed adapted to height of 1.1 m (m/s)
VHD	very hot day (-)
WMO	World Meteorological Organisation (-)
*X*cPETL_Proj_	projected future values of X cumulative physiologically equivalent temperature load (-)
Z_0_	urban roughness length (m)
α	urban surface roughness (-)
* Physiological stress acronyms from Table 1 and Table 4 excluded	
